# Are there enormous age-trends in stable carbon isotope ratios of oak tree rings?

**DOI:** 10.1177/0959683620941073

**Published:** 2020-07-15

**Authors:** Danny McCarroll, Josie E Duffy, Neil J Loader, Giles HF Young, Darren Davies, Daniel Miles, Christopher Bronk Ramsey

**Affiliations:** 1 Department of Geography, Swansea University Singleton Park, Swansea, UK; 2 Natural Resources Institute Finland (Luke), Helsinki, Finland; 3 Research Laboratory for Archaeology, Oxford University, South Parks Road, Oxford, UK

**Keywords:** dendrochronology, dendroclimatology, palaeoclimate, *Quercus*

## Abstract

We test a recent prediction that stable carbon isotope ratios from UK oaks will display age-trends of more than 4‰ per century by measuring >5400 carbon isotope ratios from the late-wood alpha-cellulose of individual rings from 18 modern oak trees and 50 building timbers spanning the 9th–21st centuries. After a very short (c.5 years) juvenile phase with slightly elevated values, the number of series that show rising and falling trends is almost equal (33:35) and the average trend is almost zero. These results are based upon measuring and averaging the trends in individual time-series; the ‘mean of the slopes’ approach. We demonstrate that the more conventional ‘slope of the mean’ approach can produce strong but spurious ‘age-trends’ even when the constituent series are flat, with zero slope and zero variance. We conclude that it is safe to compile stable carbon isotope chronologies from UK oaks without de-trending. The isotope chronologies produced in this way are not subject to the ‘segment length curse’, which applies to growth measurements, such as ring width or density, and have the potential to retain very long-term climate signals.

## Introduction

Tree rings provide unrivalled archives of annually-resolved palaeoenvironmental information and underpin the best available reconstructions of past climate (Anchukaitis et al., 2017; Luterbacher et al., 2016; PAGES hydro2k Consortium, 2017), yet they suffer from two important weaknesses. First, most of the palaeoclimate ‘proxies’ derived from tree rings rely on climatic limitation of growth. They work best where a single climate parameter is strongly growth-limiting, which tends to be summer temperature at high latitudes and altitudes (Frank et al., 2005; Fritts, 1976; McCarroll et al., 2013; Schweingruber, 1988) and moisture availability in seasonally dry areas (Cook et al., 1999). Where tree growth is not strongly limited by a single climatic control, which applies to much of the densely populated climatically-temperate mid-latitudes, tree growth proxies carry little climatic information which is often difficult to characterise and access for palaeoclimatology. Even when large numbers of samples are compiled to extract a relatively weak common signal (Cooper et al., 2013; Wilson et al., 2013), that signal may not relate to a single climatic control, and climate reconstructions thus fail established calibration and verification tests (Bothe et al., 2019; National Research Council, 2007). The second problem is that all of the tree growth proxies are influenced by tree age as well as by changes in climate, and removing the age-trend risks compromising the long-term climate signal that is often of most interest; a problem known as the ‘segment length curse’ (Cook et al., 1995). Sophisticated methods are available to deal with this but they require very high levels of replication throughout the chronology, and therefore potential loss of signal due to de-trending remains a perennial source of uncertainty (Helama et al., 2017).

Stable isotopes in tree rings have emerged as a uniquely powerful alternative to measures of tree growth, which may avoid the problems of weak climatic control and age-trends (Gagen et al., 2007; Labuhn et al., 2016; Nagavciuc et al., 2018; Li et al., 2019; McCarroll and Loader, 2004; Olson et al., 2020). Climate signals in the stable isotopes of NW European oaks, for example, growing in regions with very weak climatic control on growth, are as strong as those in the growth proxies of trees growing close to their environmental limits in northern Fennoscandia (Young et al., 2012b, 2015). In the UK, carbon isotope ratios derived from the late-wood cellulose of oaks reflect changes in summer temperature (Young et al., 2012a) and in both the UK and France oxygen isotope ratios have been linked to atmospheric circulation and thus changes in summer precipitation amount (Labuhn et al., 2016; Loader et al., 2020; Young et al., 2015). NW European oaks hold enormous potential for producing long and well-replicated isotope chronologies because very long ring width chronologies, created for dendrochronology (Baillie, 1982; Pilcher et al., 1984) and radiocarbon calibration (Becker, 1993) already exist and some of the precisely-dated wood used to build them has been archived. However, the absence of age-trends in tree ring stable isotopes has been strongly disputed (Esper et al., 2010; Helama et al., 2015; Helama and Matskovsky, 2020; McCarroll et al., 2020), and at present there is no consensus.

Unfortunately most of the information on age-trends in tree ring isotopes relates to conifers growing at high latitudes and altitudes. It is well established that carbon isotope ratios in some slow-growing conifers show a marked ‘juvenile effect’, with depressed but gradually increasing values in the rings closest to the pith, which can persist for as much as 50 years (Gagen et al., 2008; Labuhn et al., 2014). Isotope measurements are expensive, so most studies simply avoid the rings from the first few decades. In UK oak trees, by contrast, the ‘juvenile phase’ in near-pith rings of wood sampled from historic buildings lasts for only the first few rings, and it is sufficient to avoid just the five closest to the pith (Duffy et al., 2017). The absence of juvenile effects in deciduous trees has previously been reported for *Larix decidua* (Daux et al., 2011; Kilroy et al., 2016). However, since different species behave very differently with respect to juvenile trends, it is not safe to assume that any longer-term age-related trends identified in pines, for example, also apply to oak.

The best-replicated study of age-trends in carbon isotope ratios was conducted on 10-year blocks from pine trees in northern Finland (Helama et al., 2015), reporting a gentle but statistically significant age-related increase that persists for hundreds of years. This result conflicts with that of a smaller-scale but annually-resolved study based on the same species growing in the same region (Young et al., 2011a). More recently Klesse et al. (2018) have shown that young cohorts of spruce and beech trees have lower carbon isotope ratios than older cohorts growing at the same sites. However, the authors attribute the offset to differences in illumination and caution against extrapolating these findings, based on shade-tolerant species, to shade intolerant species such as oak.

Evidence for the presence or absence of age-trends in the stable carbon isotope ratios of oak tree rings is very limited. However, Brienen et al. (2017) report a correlation between isotope ratios and oak tree height, and thus age, and contend that there must be very large age-related trends, estimated at 4‰ per century, in the carbon isotope ratios of UK oaks. They conclude that ‘development trends in broadleaf species are as large as those previously assigned to CO_2_ and climate’ and that ‘credible future tree ring isotope studies require explicit accounting for species-specific developmental effects before CO_2_ and climate effects are inferred’.

It is important to stress that the age-trend in carbon isotope ratios of oak trees proposed by Brienen et al. (2017) is not an observation based on measured time-series; it is a prediction based on differences between trees. If the prediction is true, it would indeed call into question the credibility of studies using these isotopes in oaks, in the UK and probably elsewhere (e.g. Loader et al., 2008; Rinne et al., 2013; Robertson et al., 1997; Young et al., 2012a, 2015). Such an enormous age-trend would certainly compromise any environmental signals, including the independently-determined δ^13^C ‘Suess effect’ and the pooling of wood samples prior to chronology construction (Borella et al., 1998; Dorada-Liñan et al., 2011; Foroozan et al., 2019; Leavitt, 2008; Liu et al., 2015; Szymczak et al., 2012) would not be justified. We seek to test the predictions of Brienen et al. (2017) using annually-resolved stable carbon isotope time-series from UK oak timbers and trees.

## Material and methods

We present the results of more than 5400 new measurements of late-wood alpha-cellulose from individual oak tree rings, obtained from 18 modern oak trees and 50 oak building timbers, dated using ring widths and sourced from the Oxford University Dendrochronology Laboratory. The samples, from central southern England, are representative of the material available for constructing a long carbon isotope chronology for UK oak and come from the same timbers used to construct an oxygen isotope chronology used for both dating and climate reconstruction (Loader et al., 2019, 2020). The modern (post-1850 AD) and pre-industrial samples were initially analysed separately, because of the potential effects of changes in atmospheric carbon dioxide and anthropogenic changes in climate in the modern period, and then combined.

The methods we used to measure the isotope ratios are exactly those used when developing a chronology for climate reconstruction. In particular, and in contrast to Brienen et al. (2017), we only analysed the late-wood of the rings, which forms in the summer. The early-wood was not used because it forms in the spring, largely before the leaves open, and thus uses stored photosynthates (Kimak and Leuenberger, 2015; McCarroll et al., 2017; Richardson et al., 2013, 2015). The late-wood band of each ring was sliced into thin slivers using a sharp blade under magnification and each sample purified to alpha-cellulose (Loader et al., 1997), homogenised using an ultrasonic probe and freeze-dried. Samples of between 0.30 and 0.35 mg were weighed into individual silver cups and the isotope ratios measured by pyrolysis (Woodley et al., 2012; Young et al., 2011b). All chemical treatment and isotope measurements were conducted in Swansea. Results are presented in units of per mille relative to the Vienna Pee Dee Belemnite standard (δ^13^C‰). Tree rings formed after AD 1850 were corrected for changes in the isotopic ratios in atmospheric carbon dioxide (the Suess correction). No correction was made for the potential influence of increasing atmospheric carbon dioxide concentrations on tree ring isotopic composition (McCarroll et al., 2009).

We explored potential linear age-trends by fitting regression lines, with δ^13^C as a function of ring number, to the data from each tree or timber individually. These were then compiled to quantify the average trends. The statistical significance of the division of positive and negative trends was determined using the binomial test, with the r-value as a measure of effect size (McCarroll, 2017). The uncertainty of the mean slope was determined using 95% confidence intervals, so that the degrees of freedom are based on the number of trees, not the length of the series. We report results with and without the first five, potentially juvenile, rings (Duffy et al., 2017). We also checked for isotopic offsets between young and old trees, by comparing the isotopic ratios of groups of trees when they were young, with the results obtained from the same groups of trees when they were much older. Although we are specifically interested in testing for the linear age-trends that Brienen et al. (2017) have predicted, we also tested for monotonic but non-linear age-trends. Second-order polynomials were used to identify samples where more than 10% extra variance was explained by using a more flexible curve and those samples were tested for monotonicity by fitting linear regression lines to the two series halves, based on ring count.

A possible explanation for isotopic ‘age-trends’ identified in other studies, is that they are an artefact of the methods that are most commonly used to define them (Duffy et al., 2019; McCarroll et al., 2020). We test that possibility by replacing the pre-industrial timber results, used in this study, with their mean values, so that the differences in length and offsets are preserved but all of the series have zero trend and zero variance.

## Results

**Figure 1.**
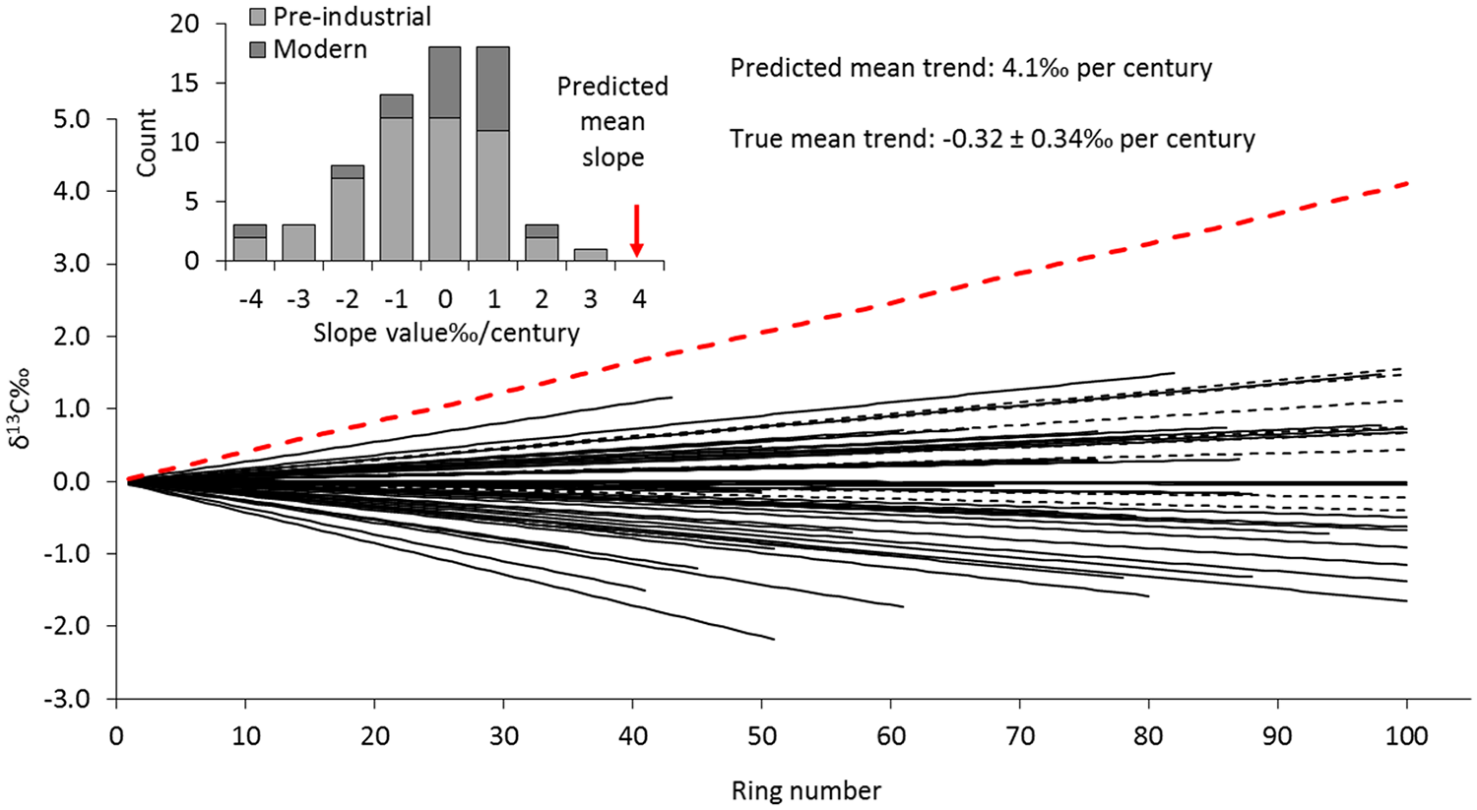
Linear trends of carbon isotope ratio plotted against ring number for 18 modern oak trees (dashed black lines) and 50 pre-industrial oak timbers (solid black lines) compared with the mean trend predicted by Brienen et al. (2017) (dashed red line). Inset shows the distribution of slope values.

Of the 18 modern trees, including the first five (near-pith) rings, six show a decline with ring number and 12 show an increase (Figure 1). Assuming that age-trends are random, the probability of 12 from 18 falling in the same direction is about 25% (binomial test: *p* = 0.24, effect size *r* = 0.28). The range of the 18 trends (slope of the regression line) is +0.016 to –0.036‰/year and the mean is 0.001 ± 0.006‰/year. The mean trend is thus very weak and far from statistically significant. The means of the 18 series fall within a range of 2.5‰ (–23.67 to –26.11). After removing the first five rings, which tend to have high values, the division of positive and negative slopes is the same and the range is similar (+0.018 to –0.034‰/year), the mean slope is slightly steeper (0.002 ± 0.006‰/year) but still far from statistically significant.

For the 50 pre-industrial timbers, when the first five rings are included the division between positive and negative slopes (20:30) is not statistically significant (*p* = 0.20, *r* = 0.18), the range of slope values is +0.027 to –0.043‰/year and the mean slope has confidence limits that do not cross zero (–0.005 ± 0.004‰/year). However, whereas the high juvenile values in the modern samples served to mute the overall small average increase, in the pre-industrial samples they serve to amplify the weak decline. When the first five rings are removed, the range of slopes is +0.020 to –0.048‰/year and the average slope drops to –0.003 ± 0.004‰/year and is no longer statistically significant.

When the modern and pre-industrial trees and timbers are combined, to give a sample of 68 time-series, the split of positive and negative slopes is almost equal (32:36) and the average trend with ring number is negative but not significantly different from zero (–0.003 ± 0.003‰/year). After excluding the first five rings the results are even closer to zero slope (33:35, –0.002 ± 0.004‰/year).

After removing the first five rings there were 22 cases where a second-order polynomial increased the explained variance by more than 10%. Of those only two were monotonic, with one (PI 36) falling gently and then more steeply and the other (M15) falling steeply and then more gently. Of the remainder, 11 showed a fall and then a rise in δ^13^C with ring number and nine showed a rise and then a fall. It is clear that there are no consistent non-linear trends with ring number in these samples.

We tested for an offset, due to age, by aligning the trees and timbers by ring number and comparing the values obtained when a group of trees was young, with the values obtained from the same group of trees when they were older. In the total data set there are 40 trees with at least 79 rings, so we compare the mean isotope value obtained from 40 trees for rings 10 to 29, with the mean value obtained for rings 60 to 79, and they are almost identical (–24.68 ± 0.31 and –24.68 ± 0.27). The δ^13^C values obtained from the first five rings are slightly, but not significantly, higher (–24.44 ± 0.24). There are 30 trees with at least 94 rings so we compare the mean values from rings 10 to 29 (–24.64 ± 0.24), with those for rings 75 to 94 (–24.62 ± 0.35), and again they are almost identical. There is no evidence for offsets in δ^13^C caused by differences in age.

**Figure 2.**
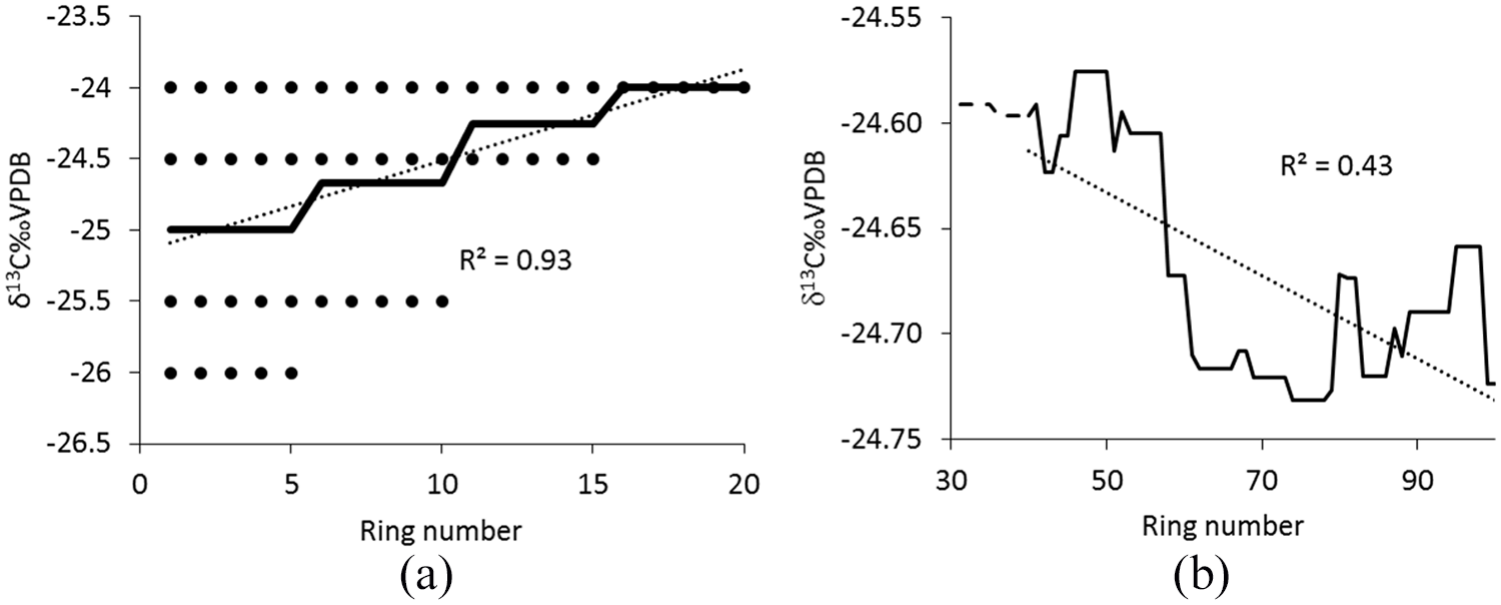
Simple illustration (a) of how spurious trends are produced when averaging the isotopic values of four series that are completely flat (black dots) but are offset and of different length. When the pre-industrial series used in this study are replaced by their mean values, so that all 50 series have zero trend and zero variance, the average shows a strong but completely spurious trend (b).

For the pre-industrial timbers used in this study, the overall mean values of the series vary by almost 3‰ (–25.90‰ to –22.95‰), and although 48 series have at least 40 rings, only 11 reach 100 rings. When each of the series is replaced by the mean value, so that for example a timber with 50 rings and a mean of –23.0‰ is replaced with 50 identical values of –23.0‰, then of course the mean of the trends will be zero, because all of the constituent series have a trend of zero and zero variance. When these flat series are compiled by ring number and the average isotope value is plotted (Figure 2b), the average over the period with constant replication is flat with zero variance, as expected. However, as soon as series begin to drop-out, the mean series adopts a trend and shows considerable variability. In this case, over the section with changing replication (40–100 rings), the ‘age-trend’ produced by the ‘slope of the mean’ approach appears to explain 43% of the variance, even though the true mean trend is zero and the individual series have zero variance.

## Discussion

The enormous positive age-trend in oak tree ring δ^13^C, predicted by Brienen et al. (2017) clearly does not exist. Of the 68 trees and timbers included in this study not one has such a strong rising trend with ring number and the average trend is not significantly different from zero. After removing five rings closest to the pith the average slope has less than 4% of the magnitude of that predicted by Brienen et al. (2017) and is in the opposite direction.

Brienen et al. (2017) sampled the outermost five rings from 78 trees from a single English forest, converted the five-year blocks into cellulose and measured the isotope ratios. The range of values they obtained, however, is more than 7‰. For comparison the individual values from this study were re-calculated to represent all possible (>5000) mean values for five consecutive rings. The total range is only 5.2‰, which includes samples from the depths of the Little Ice Age to the peak temperatures of the 21st century, sampled from across central southern England (Loader et al., 2019). The lowest value for an individual late-wood ring in this study is –28.05‰ and the lowest mean value for a group of five consecutive rings is –27.37‰. In contrast, almost half of the values reported by Brienen et al. (2017) fall below –28‰ with several well below –30‰. Combining several years into a single sample should reduce the variance of a time-series, and reported difference in δ^13^C between late-wood and early-wood are small (Kimak and Leuenberger, 2015), so the reason for the very large range and extremely low values reported by Brienen et al. (2017) remain unexplained, but it is clear that they do not resemble the δ^13^C values obtained for late-wood cellulose time-series of UK oaks.

The conclusions of Brienen et al. (2017) are also based upon the assumption that variations across space can be translated directly into variations through time, which in all branches of Quaternary science is a dangerous assumption. The fact that saplings growing in deep shade give lower carbon isotope ratios than tall trees growing in full sun is not surprising, since in the absence of strong moisture stress it is photon flux that dominates fractionation (McCarroll and Loader, 2004). However, it is not reasonable to assume that saplings growing in deep shade provide a reliable proxy for the juvenile years of oak trees that live to reach maturity and become a source of timber. Oaks are shade intolerant trees, that grow to be very large and can be very long-lived, so the percentage of saplings that live to reach maturity must be very small indeed. Most are doomed to die for lack of light. In such circumstances space does not provide a good substitute for time, and if the aim is to characterise age-trends in mature oaks and timbers, then the only suitable methodology is to measure those trends. If the conclusions are to be used to make recommendations about the interpretation of oak δ^13^C chronologies, then the measurement protocols need to be comparable to those that are actually used to build those chronologies. For oaks, that means the alpha-cellulose of late-wood not from trees sourced from single-age stands where stand-development may introduce community-related trends, but from within naturally-regenerating, mature woodland.

Brienen et al. (2017) support their argument for age-trends in tree ring δ^13^C by presenting the results of the large set of pine samples, previously reported by Helama et al. (2015). The reported trend in these samples is, however, more than an order of magnitude weaker than that predicted for the broadleaf trees, with a rise of less than 0.004‰/year. Although Helama et al. (2015) interpret this as an age-related trend, which may be true, it could reflect orbitally-forced climate change at high latitudes (Esper et al., 2012; McCarroll et al., 2013), with falling temperatures driving an expansion of the zone of Arctic high pressure, leading to more sunny conditions (Gagen et al., 2011; Loader et al., 2013; Young et al., 2019), which would result in higher (less depleted) carbon isotope ratios. Alternatively, the gentle rising trend reported by Helama et al. (2015) may be an artefact of the ‘trend of the mean’ approach, which frequently results in spurious age-trends (Duffy et al., 2019).

## Conclusions

We have presented more than 5400 δ^13^C results from the late-wood alpha-cellulose of the individual rings of 18 modern trees and 50 pre-industrial timbers drawn from across central southern England and spanning the 9th–21st centuries. It is clear these samples, which are representative of the material available for chronology construction, do not display consistent age-trends. The division of positive and negative trends is almost even and the mean trend, ignoring the five rings closest to the pith, is very close to zero. We demonstrate this by measuring the trends in individual trees, and also by comparing the isotope values obtained from groups of trees when they were young with isotope values from the same groups of trees when they were old.

We conclude that there are no consistent age-trends in the oak samples available for tree ring stable isotope chronology construction in the UK. The trends that are present in individual timbers are either climatic signals, which will be enhanced by replication, or random noise that will be averaged out. The enormous positive age-trend in oak tree ring δ^13^C, predicted by Brienen et al. (2017) clearly does not exist.

## Supplemental Material

McCarroll_et_al_supp – Supplemental material for Are there enormous age-trends in stable carbon isotope ratios of oak tree rings? Click here for additional data file. Supplemental material, McCarroll_et_al_supp for Are there enormous age-trends in stable carbon isotope ratios of oak tree rings? by Danny McCarroll, Josie E Duffy, Neil J Loader, Giles HF Young, Darren Davies, Daniel Miles and Christopher Bronk Ramsey in The Holocene
